# Interesting Facts Connected with the Animal Kingdom; with Some Remarks on the Unity of Our Species

**Published:** 1842-07

**Authors:** 


Art. VII.—*Interesting Facts connected with the Animal Kingdom; with some Remarks on the Unity of our Species.* By John C. Hall, m.d. —*London,* 1841. 8vo, pp. 301.

The author informs us in his preface that this work contains "the substance of a course of lectures on the animal kingdom, delivered at many of the scientific institutions of the metropolis." These lectures doubtless were well suited for their purpose, and proved both useful and instructive to the hearers. In their present form, however, they contain nothing novel; nor do we find in them old knowledge put in a new form. Nevertheless, such readers as are unacquainted with Dr. Prichard's great work, and who are desirous of obtaining general views of the animal kingdom, and a compendium of the natural and physical history of man, may peruse this little volume with both pleasure and profit.

